# Childhood Asthma in Saudi Arabia: Insights from a Meta-Analysis on Its Prevalence

**DOI:** 10.3390/children11121550

**Published:** 2024-12-20

**Authors:** Abdullah Alzayed

**Affiliations:** Department of Pediatrics, College of Medicine, Imam Mohammad Ibn Saud Islamic University (IMSIU), Riyadh 13317, Saudi Arabia; aaalzayed@imamu.edu.sa

**Keywords:** asthma, children, Saudi Arabia, prevalence, meta-analysis

## Abstract

A comprehensive review and meta-analysis were conducted with the purpose of determining the extent to which asthma is prevalent among children in Saudi Arabia. This was done with the intention of addressing the dearth of data at the national level regarding this significant health concern. The study included data from PubMed, Embase, Cochrane Library, and Google Scholar for the period 2015–2024, focusing on studies that reported the prevalence of asthma among children in the country. The primary outcome was the pooled prevalence of physician-diagnosed asthma among children. Eight studies, comprising a total of 9454 children, were included in the analysis. The estimated pooled prevalence of asthma was 28.9%, with a higher prevalence observed among boys compared to girls. A random-effects model was used to account for heterogeneity among studies, which was notably high (I^2^ = 99%). Factors contributing to the high prevalence included increasing urbanization, air pollution, and specific environmental exposures, particularly in regions like Jazan. Limitations of the analysis included reliance on self-reported data without spirometric confirmation and potential publication bias. Despite these challenges, the findings emphasize the urgent need for public health interventions to reduce asthma prevalence and improve outcomes. Future research should incorporate standardized diagnostic methods and objective measurements to provide more accurate estimates and to develop effective management strategies.

## 1. Introduction

Over the years, there has been a notable rise in the frequency of allergy disorders diagnosed by physicians. These diseases today impact between 10% and 30% of the world’s population [1]. The pediatric allergic illnesses include atopic dermatitis, allergic rhinitis, food allergies, and asthma, which is the most prevalent chronic condition affecting both children and adults [2]. Asthma is the most common inflammatory disease in children and a serious public health concern. Chest tightness, wheezing, shortness of breath, and repeated coughing are mild-to-severe symptoms of extensive and varying airway blockage caused by inflammation [3,4].

Asthma imposes significant financial strain on patients, their families, and the healthcare system. In addition to causing persistent lung function decrease, asthma can seriously impair one’s capacity to conduct regular daily activities, such as sports and outdoor activities, sleep disturbances, and exhaustion [5,6]. Over 5.3 million school days are missed annually due to it, and it is the third-most common reason why children are admitted to hospitals [7].

Research aimed at determining the prevalence of asthma in Saudi Arabian children has yielded varying results, ranging from 8% to 25% [8]. The study environment, participant characteristics, and the variety of methods employed to assess the incidence of asthma could all be contributing factors to this vast variance in prevalence [9]. Based on how strict each study’s methodology and data-analysis strategy were, the quality of each study likewise varied considerably from the other. Numerous studies conducted in Saudi Arabia have qualitatively evaluated the prevalence of asthma in children, and these evaluations were predicated on different asthma definitions and diagnostic standards [10,11,12].

In order to provide a comprehensive picture of this crippling illness among Saudi Arabian children, a quantitative analysis was, therefore, deemed required. Therefore, in order to estimate the pooled prevalence of asthma among children in Saudi Arabia, this study was conducted as a protocol for a systematic review and meta-analysis. The review question that guided the development of this meta-analysis was as follows: Can you provide an estimate of the overall prevalence of asthma in Saudi Arabia, considering the most recent data?

## 2. Materials and Methods

### 2.1. Protocol and Registration

This meta-analysis has been conducted in accordance with the Preferred Reporting Items for Systematic Reviews and Meta-Analyses (PRISMA) statement [13], and the protocol for this meta-analysis has been properly recorded in PROSPERO (CD 4217239828), with assertions based on the Meta-analysis of Observational Studies in Epidemiology to direct this research [14]. This meta-analysis neither includes any attempts to obtain unpublished data nor modify the methodology described in the clinical trials under analysis, and, thus, it does not use patient data. Hence, there was no attempt to obtain informed consent. Since this study is a review that uses published data from the included studies, the Institutional Review Board waived the requirement for ethical approval.

### 2.2. Search Strategy

In order to gather the most recent research on the subject while remaining current, a thorough literature search was conducted to find pertinent studies published between 2015 and 2024. Medline via PubMed, Embase, Cochrane Library, and Google Scholar, regardless of language, as well as relevant translations, were employed. To identify potential articles for this review, medical subject headings (MeSH) and EMBASE subject headings were utilized and are detailed in the search strategy steps.

The search strategy was tailored to each database and was based on the review questions for this meta-analysis study. The search strategy combined terms related to four areas: (1) population: infant/children between the ages of 1 to 18 years, (2) exposure: asthma, (3) prevalence, and (4) Saudi Arabia. “Prevalence” or “epidemiology” (title) was coupled with “wheeze” or “asthma symptoms” (title), along with “infant”, “children”, “preschool” (subject), “asthma”, “bronchial wheezing”, “respiratory wheezing”, and “Saudi Arabia” as search phrases. The Boolean search function “and” was utilized to combine the search terms. In addition, the reference sections of the recovered original articles and reviews were carefully examined to ensure that no relevant studies were missed.

### 2.3. Inclusion and Exclusion Criteria

The inclusion criteria for this meta-analysis were bounded by the PECO format, where the (P) population under review was children categorized as 1–18 years old, the exposure (E) was asthma, the comparator (C) was none (as this study did not call for a comparison of the exposure), and the (O) outcome of interest was the prevalence of asthma among children living in Saudi Arabia. After doing an initial examination of the abstracts and titles, the following criteria were utilized to determine whether or not a study was eligible. These are the original cross-sectional studies that addressed the following subjects: (1) reported the prevalence of asthma in Saudi Arabia; (2) examined school-based or population-/community-based settings; and (3) estimated the prevalence of asthma using The International Study of Asthma and Allergies in Childhood (ISAAC) [15] or short-form questionnaires that study participants or their parents completed, and (4) it was possible to determine the prevalence of asthma using the outcome data from the studies.

Research populations older than 12 years, animal studies, abstracts, letters, review papers, editorials, and case reports were the only resources that were not evaluated for inclusion.

### 2.4. Data Extraction

The retrieved records from the databases were screened for the titles and abstracts. For the full-text review, only abstracts that met the selection criteria were selected. Uncertainties about the choice of studies were cleared up after consulting with a subject-matter expert. Duplicates were removed after confirming the most recent and comprehensive version. Additional sources were the extracted studies’ reference lists. The full-text studies that were retrieved underwent additional evaluation to verify if they met the inclusion requirements. Relevant data fields from the selected full-text studies were extracted and recorded in a spreadsheet data collection form. Each study’s author information, publication year, study location, methodology, age range, sample size, gender, physician-diagnosed asthma, asthma-assessment instrument, and stated prevalence of asthma were obtained from the data.

### 2.5. Quality Assessment

This study used the National Institutes of Health Quality Assessment Tool for Observational Cohort and Cross-Sectional Studies [16], assessing 14 criteria for the in-study methods or implementation. The scoring was done on a scale of “No”, “Yes”, “Cannot Be Determined”, “Not Applicable”, and “Not Reported”. The risk of publication bias was visually evaluated by Begg’s modified funnel plot [17].

### 2.6. Data Synthesis and Statistical Analysis

The precision of the summary estimate, which contained summary estimates of the prevalence of asthma in children, was evaluated with the help of a confidence interval (CI) from 95% to determine how accurate it was. Using the prevalence and sample size from all of the included studies, we were able to determine the standard error of the prevalence. We built and displayed forest plots to show the prevalence with a 95% confidence interval. The statistical analyses for the meta-analysis were conducted using STATA version 14.0 (STATA Corporation, College Station, TX, USA) and R version 3.3.0 (R Foundation for Statistical Computing, Vienna, Austria). When the *p*-value was less than 0.05, statistical significance was highlighted. A weighted random-effects model based on the variance’s inverse was employed.

The heterogeneity was assessed using the I^2^ statistic. By visually examining the funnel plot, publication bias was determined, and Egger’s test analysis was conducted on the basis of gender and provincial representation in Saudi Arabia to examine the observed heterogeneity. Following the removal of one sizable national-level study, sensitivity analysis was performed to evaluate the changes in pooled estimates.

### 2.7. Outcome

The primary outcome analyzed in this meta-analysis study was the prevalence of asthma in children residing in Saudi Arabia.

### 2.8. Other Outcomes

The prevalence of asthma among children based on the regional presentation was included as another outcome of interest in this review study.

### 2.9. Subgroup Analysis

We created forest plots independently according to gender for subgroup analysis.

## 3. Results

### 3.1. Study Search

**Figure 1:**
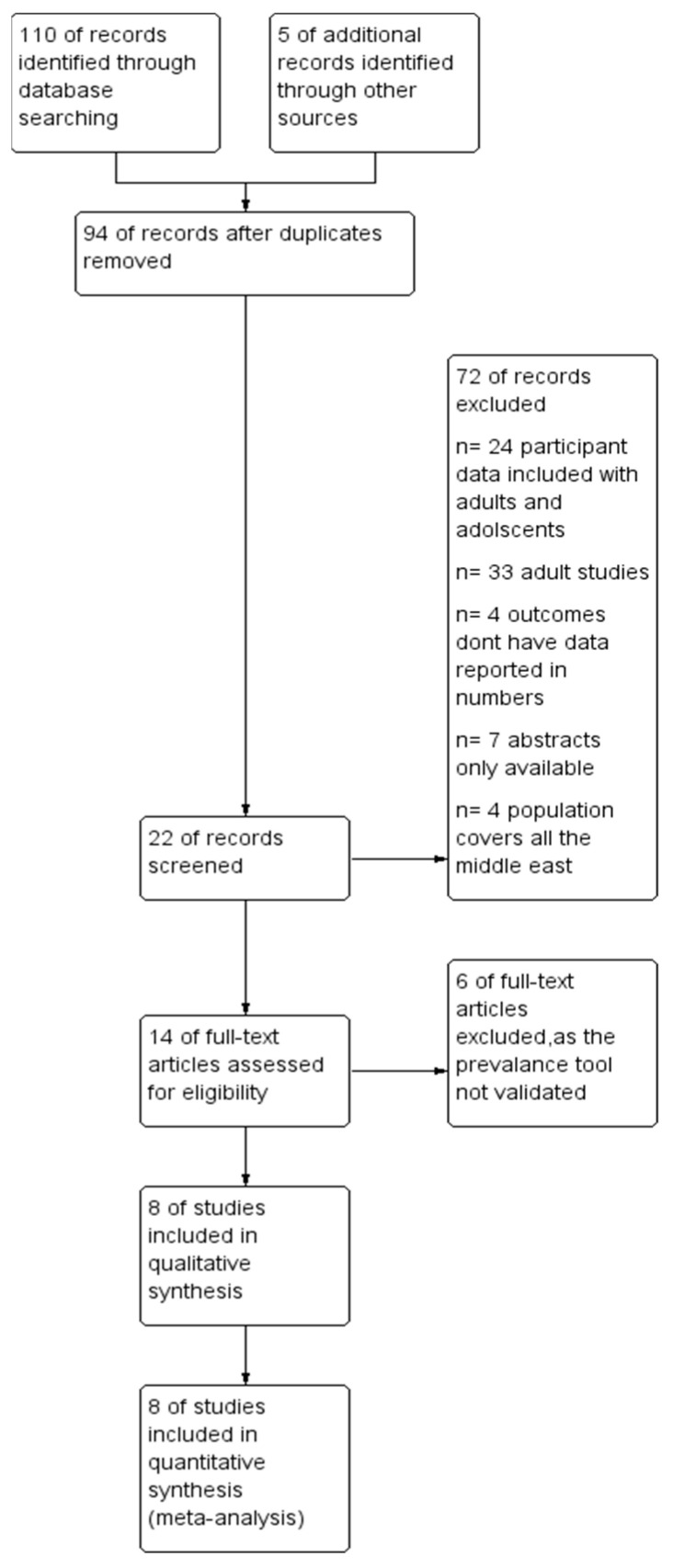
Prisma flow chart of selection of studies for meta-analysis.

A preliminary search yielded 115 studies from electronic databases. Following the removal of duplicates (21 studies), 94 studies were screened according to the selection criteria based on their titles and abstracts. Twenty-two eligible abstracts were selected for full-text screening. The meta-analysis included eight studies that fulfilled the inclusion criteria. You can see the search process for this study in Figure 1, which is a PRISMA flow diagram [18].

### 3.2. Characteristics of Studies Included in the Meta-Analysis

There were 9452 children from the eight included studies [19,20,21,22,23,24,25,26] that were included in this study. Four of the eight investigations were conducted in rural areas [19,22,25,26], whereas the remaining four involved urban populations. All selected studies were cross-sectional, and the age group of the participants ranged from 1 to 12 years, with a mean of 8.3 years. To identify asthma, six studies used the (ISAAC) tool [16], one study [23] used the modified ISAAC tool, and one study [20] used the short-form questionnaire. Using a straightforward random sample method, each of the included researchers recruited the participants. Six of the studies that were included in the analysis reported the prevalence of asthma in different gender categories, in addition to providing a summary estimate.

Only one study [19] mentioned the research’s timing in the study. The sample population ranged from 143 to 3817. Table 1 displays the features of the studies that were part of the meta-analysis.

### 3.3. Quality Assessment

This study used the National Institutes of Health Quality Assessment Tool for Observational Cohort and Cross-Sectional Studies, assessing 14 criteria for the in-study methods or implementation. Based on these criteria, none of the studies has an excellent score, and all the eight studies were rated between fair and good quality. The quality assessment tool for each selected study is presented in Table 2.

### 3.4. Publication Bias

**Figure 2:**
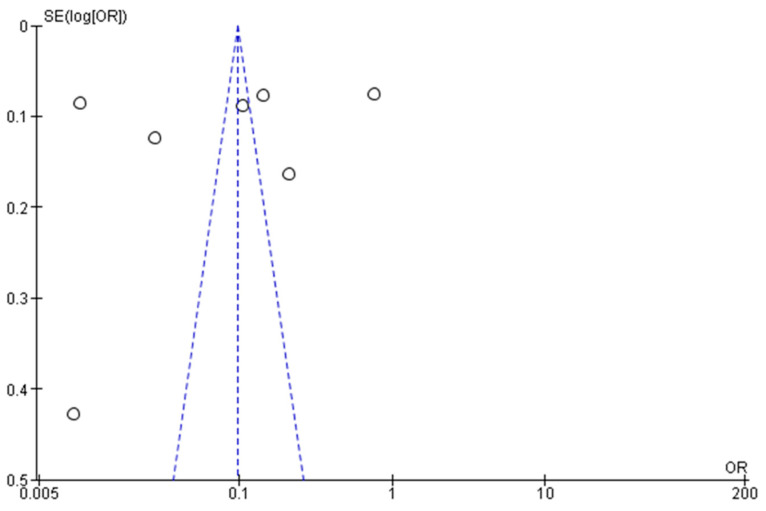
Publication bias funnel plot.

The publication bias was assessed for the primary outcome (prevalence of asthma among children residing in Saudi Arabia) by visual inspection of the funnel plot and statistically by Begg’s modified funnel plot, as presented in Figure 2, with the Egger’s test t = 1.265 and *p*-value = 0.22. For the pooled meta-analysis for the prevalence of asthma among children residing in Saudi Arabia, Egger’s test and funnel plots revealed significant publication bias.

### 3.5. Primary Outcome

#### 3.5.1. Meta-Analysis of the Prevalence of Asthma Among Children Living in Saudi Arabia

**Figure 3:**
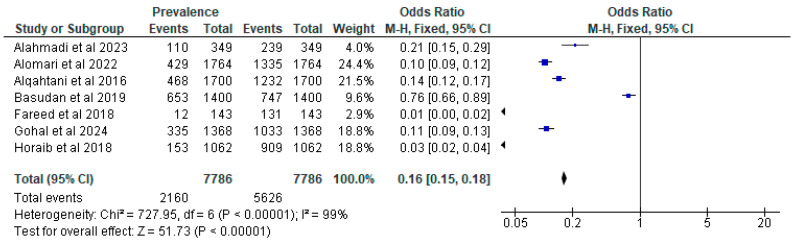
Forest plot illustrating the meta-analysis for the prevalence of asthma among children residing in Saudi Arabia [19,20,21,22,23,25,26].

Among the total of eight studies, the prevalence diagnosed by a physician was considered. Among the eight studies, the lowest-recorded prevalence of asthma in children was 0.8% [19], while the highest prevalence reached 46.64% [20]. When looking at the prevalence of asthma among children, the pooled estimate using a random-effects model was 0.16 [0.15, 0.18]. Heterogeneity testing showed that I^2^ = 99%, with Chi^2^ = 727.95 and df = 6. The *p*-value is less than 0.00001. Figure 3 shows the forest plot, which shows this estimate.

#### 3.5.2. Meta-Analysis of the Prevalence of Asthma Based on Provinces in Saudi Arabia

**Figure 4:**
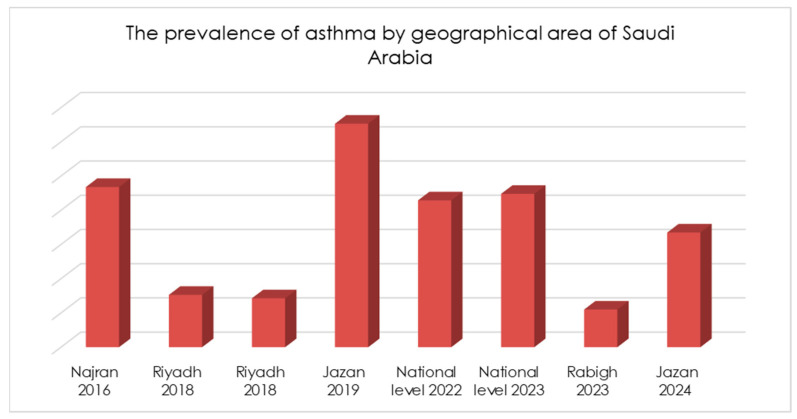
The prevalence of asthma from the included studies by geographical area of Saudi Arabia.

From all the provinces of Saudi Arabia, the included studies had a representation of the population only from five provinces. The comparison of the prevalence of asthma among the children from these provinces is displayed in Figure 4. The highest prevalence of asthma in children was noted in the Jazan province, ignoring the cumulative number from studies at the national level. While variations in living conditions, allergens, and socioeconomic status between urban and rural areas were associated with differences in findings, these differences were not statistically significant and may not be clinically meaningful.

**Figure 5:**
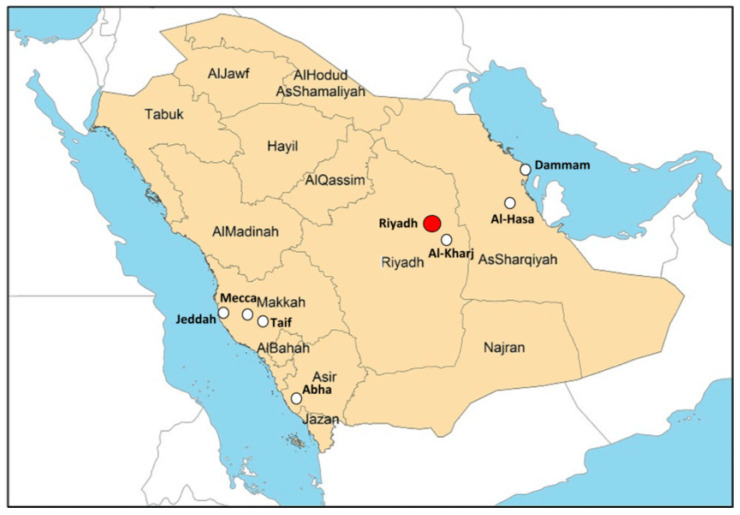
Saudi Arabia’s geographic landscape: a visual overview.

**Figure 6:**
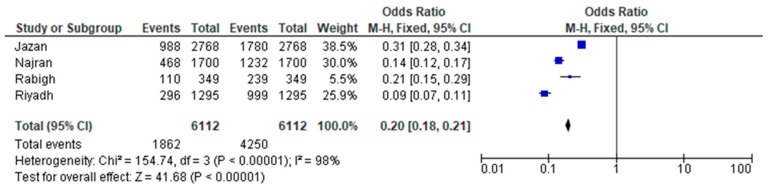
Forest plot of the meta-analysis for the prevalence of asthma by provincial representation.

Figure 5 provides a visual representation of Saudi Arabia’s diverse geography, showcasing its 13 provinces and the key cities/regions relevant to this discussion After considering the representation from different provinces, the combined estimate for the prevalence of asthma among children was 0.20 [0.18, 0.21]. The heterogeneity test indicated Chi^2^ = 154.74, df = 3 (*p* < 0.00001); I^2^ = 98%. The forest plot illustrated in Figure 6 represents this estimate. Figure 6 illustrates the aggregated findings of the meta-analysis concerning the prevalence of asthma across different provinces.

This meta-analysis revealed striking regional disparities in childhood asthma rates across Saudi Arabia. These variations underscore the influence of environmental factors such as climate, altitude, and geographic features. The unique geography of Jazan, with its diverse terrain and significant coastal population, may contribute to its high asthma prevalence.

### 3.6. Subgroup Analysis

#### 3.6.1. Gender-Based Prevalence of Asthma

**Figure 7:**
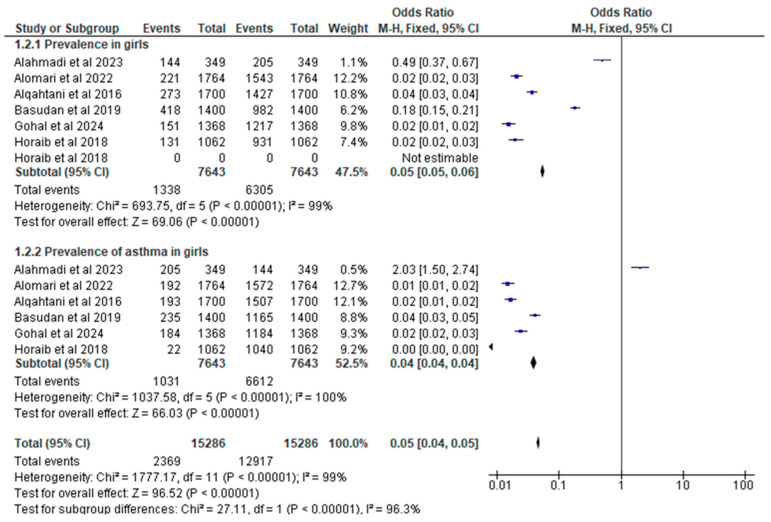
Forest plot of meta-analysis of asthma prevalence by gender (school-based studies) [19,20,22,23,25,26].

All eight studies provided data on the gender-specific prevalence of asthma. The incidence of asthma in school-based studies among boys and girls was 14.16%. Also, 0.05 [0.05, 0.06] Chi^2^ = 693.75, df = 5 (*p* < 0.00001); I^2^ = 99% and 10.91% 0.04 [0.04, 0.04] Chi^2^ = 1037.58, degrees of freedom = 5 (*p* < 0.00001); I^2^ = 100%. The results of this investigation did not reveal any signs of a decrease in heterogeneity within this particular subgroup. As shown in Figure 7, there was not a discernible difference in the severity of heterogeneity between the studies based on the gender of the participants. Based on the findings of this study, Table 3 provides a thorough summary of the data on the prevalence of asthma among children living in Saudi Arabia.

In addition to socioeconomic factors, exposure to passive smoking and environmental pollutants may contribute to the increased risk of asthma among boys.

#### 3.6.2. Sensitivity Analysis

The sensitivity analysis was performed to assess the robustness of the meta-analysis results. Sensitivity analysis involved the exclusion of two studies [21,23] that included participants mixed with adolescents and had a sample size of 143. This exclusion revealed no significant change in the prevalence of asthma, recorded at 0.18 [0.18, 0.19]. Chi-squared statistic = 702.12, degrees of freedom = 6 (*p* < 0.00001); I-squared = 99%.

The results of the sensitivity analysis indicated that the exclusion of these studies did not substantially alter the pooled prevalence estimate. This suggests that the overall findings of the meta-analysis are robust and not overly influenced by these specific studies. However, the high level of heterogeneity among the included studies, as indicated by the I^2^ value of 99%, remains a significant concern. This heterogeneity suggests substantial variability in the prevalence of asthma across different populations and settings. The sensitivity analysis, by addressing potential sources of bias and methodological limitations, strengthens the credibility of the meta-analysis findings and provides valuable insights into the factors contributing to this heterogeneity.

## 4. Discussion

The meta-analysis conducted on the prevalence of asthma among children in Saudi Arabia provides important insights into the scope and dynamics of this chronic condition within the country. The pooled prevalence estimates of asthma, found to be 28.9%, is notably higher than some global prevalence figures, which typically range between 10–20%, depending on the region and age group studied [27]. The study’s findings resonate with the estimated prevalence of asthma as 8–25% among children in Saudi Arabia [28].

The observed high prevalence can be attributed to several factors, such as increasing urbanization, air pollution, climatic factors, and changes in lifestyle, which might contribute to higher susceptibility to asthma among Saudi children. Saudi Arabia, with its unique environment characterized by an arid climate, extreme temperatures, and dust storms, can exacerbate respiratory conditions like asthma [29]. Moreover, regional differences noted in the prevalence, with particularly high rates in provinces such as Jazan, further emphasize the impact of local environmental factors. Jazan, for instance, is a coastal region, which may have higher exposure to humidity and allergens, factors known to exacerbate asthma symptoms [30]. The other asthma risk factors typically arise from air pollution and poor air quality, along with socioeconomic factors.

The gender-based subgroup analysis revealed that the prevalence of asthma was higher in boys (14.16%) compared to girls (10.91%), which is consistent with trends reported in other studies globally [31]. The biological and hormonal differences between boys and girls, alongside possible variations in exposure to environmental allergens or irritants, may explain these disparities [32]. Boys generally have smaller airways compared to girls in early childhood, which could increase their risk of developing respiratory conditions like asthma [33]. However, such gender differences tend to equalize as children grow older, suggesting the need for age-specific asthma management strategies.

### Limitation

A significant limitation of the studies included in this meta-analysis is the reliance on self-reported questionnaires, such as the ISAAC tool, without objective diagnostic confirmation, like spirometry. This could lead to overestimation or underestimation of asthma prevalence due to misclassification. Spirometry, although challenging to perform in young children, remains a gold standard for asthma diagnosis and should ideally be incorporated in future studies along with the ISAAC tool to enhance diagnostic accuracy [34]. The absence of spirometry in the included studies is acknowledged as a limitation, highlighting a gap that future epidemiological studies must address for more reliable data. Thus, to clearly show the prevalence of asthma in children, studies should be undertaken with questionnaire tools, clinical data, and relevant tests.

The meta-analysis also indicated substantial heterogeneity among the included studies (I^2^ = 99%). The variations could stem from differences in study design, sampling methods, definitions of asthma, and even socio-economic conditions across regions. This high heterogeneity limits the ability to generalize findings across all Saudi Arabian children effectively. Sensitivity analysis, which attempted to reduce this variability by excluding studies with mixed-age participants, still showed considerable heterogeneity, indicating that intrinsic differences between study populations and methodologies remain influential factors.

Publication bias was another potential limitation identified through visual inspection of the funnel plot and statistical tests, which indicated asymmetry. This suggests that smaller studies reporting lower prevalence might be underrepresented in the literature, potentially skewing the overall prevalence estimate upward. The bias could also reflect a greater tendency for studies with significant findings to be published compared to those with non-significant outcomes [35].

## 5. Conclusions

According to the results of this meta-analysis, children living in Saudi Arabia have a significant prevalence of asthma compared to global prevalence and prevalence in neighboring countries. To fully represent the trend in the prevalence of childhood asthma, national-level estimates from all 13 provinces of Saudi Arabia are required. On correctly identifying the prevalence and risk factors, asthma control interventions must begin at the primary healthcare level, where patients can receive proper diagnosis and management through community education, prioritization of necessary medications and equipment, and medical staff training.

## Figures and Tables

**Table 1:** Attributes of studies and the prevalence of asthma among children in Saudi Arabia included in the meta-analysis.

Author Name	Year	City (Province)	Study Setting	Sample Size	Tool Used	Prevalence in Girls		Prevalence in Boys		Prevalence in Children	
						*n	%	*n	%	*n	%
Alqahtani et al. [19]	2016	Najran	Cross-sectional	1700	ISSAC	193	22.7	273	32.3	468	27.5
Horaib et al. [20]	2018	Riyadh	Cross-sectional	1062	Short Form	22	14.4	131	12.4	153	14.4
Fareed et al. [21]	2018	Riyadh	Cross-sectional	143	ISSAC	NA	NA	NA	NA	143	0.8
Basudan et al. [22]	2019	Jazan	Cross-sectional	1400	ISSAC	235	16.7	418	29.8	653	46.64
Alomari et al. [23]	2022	Saudi Arabia	Cross-sectional	1764	Modified ISSAC	192	10.9	221	11.9	429	11.9
Aleid et al. [24]	2023	Saudi Arabia	Cross-sectional	1666	ISSAC	J16	NA	NA	NA	448	26.9
Alahmadi et al. [25]	2023	Rabigh	Cross-sectional	349	ISSAC	205	58.7	144	41.3	110	31.5
Gohal et al. [26]	2024	Jazan	Cross-sectional	1368	ISSAC	184	13.5	151	11	335	24.5
Total				9452		1031	10.91	1338	14.16	2739	28.98

ISSAC: The International Study of Asthma and Allergies in Childhood; * Reported as cumulative prevalence in the article; NA = Not available.

**Table 2:** Quality assessment of included studies (NIH Quality Assessment tools).

Criteria	Alqahtani et al. [19]	Horaib et al. [20]	Fareed et al. [21]	Basudan et al. [22]	Alomari et al. [23]	Aleid et al. [24]	Alahmadi et al. [25]	Gohal et al. [26]
1. Was the research question or objective in this paper clearly stated?	YES	YES	YES	YES	YES	YES	YES	YES
2. Was the study population clearly specified and defined?	YES	YES	YES	YES	YES	YES	YES	YES
3. Was the participation rate of eligible people at least 50%?	YES	YES	YES	YES	YES	YES	YES	YES
4. Were all the subjects selected or recruited from the same or similar populations (including the same time period)? Were inclusion and exclusion criteria for being in the study prespecified and applied uniformly to all participants?	YES	YES	CD	NO	NO	YES	NO	NO
5. Was sample size justification, power description, or variance and effect estimates provided?	NO	YES	CD	NO	YES	YES	NO	YES
6. For the analyses in this paper, were the exposure(s) of interest measured prior to the outcome(s) being measured?	NO	NO	NO	NO	YES	YES	YES	YES
7. Was the timeframe sufficient so that one could reasonably expect to see an association between exposure and outcome if it existed?	CD	CD	CD	CD	CD	CD	CD	CD
8. For exposures that can vary in amount or level, did the study examine different levels of exposure as related to the outcome (e.g., categories of exposure, or exposure measured as continuous variable)?	YES	YES	YES	YES	YES	NO	CD	YES
9. Were the exposure measures (independent variables) clearly defined, valid, reliable, and implemented consistently across all study participants?	YES	YES	YES	YES	YES	NO	NO	YES
10. Was the exposure(s) assessed more than once over time?	NO	NO	NO	NO	NO	NO	NO	NO
11. Were the outcome measures (dependent variables) clearly defined, valid, reliable, and implemented consistently across all study participants?	YES	YES	CD	YES	YES	CD	YES	YES
12. Were the outcome assessors blinded to the exposure status of participants?	NO	NO	NO	NO	NO	NO	NO	NO
13. Was the loss to follow-up after baseline 20% or less?	NA	NA	NA	NA	NA	NA	NA	NA
14. Were key potential confounding variables measured and adjusted statistically for their impact on the relationship between exposure(s) and outcome(s)?	NR	NR	NR	NR	NR	NR	NR	NR
Quality of studies	GOOD	GOOD	FAIR	FAIR	GOOD	FAIR	FAIR	GOOD

CD: cannot be determined, NA: not applicable, NR: not reported.

**Table 3:** Overall results of the prevalence of asthma in children living in Saudi Arabia.

Item	Prevalence %	*p* Value	*I* ^2^	OR
Children	28.98	<0.001	99	0.16 [0.15, 0.18]
Girl	10.91	<0.001	99	0.05 [0.05, 0.06]
Boy	14.16	<0.001	99	0.05 [0.04, 0.05]
